# The Levels and Duration of Sensory and Motor Blockades of Spinal Anesthesia in Obese Patients That Underwent Urological Operations in the Lithotomy Position

**DOI:** 10.1155/2015/453939

**Published:** 2015-04-29

**Authors:** Taner Ciftci, Ali Bestemi Kepekci, Hatice Pınar Yavasca, Hayrettin Daskaya, Volkan İnal

**Affiliations:** ^1^Department of Anesthesiology and Reanimation, Dicle University Medical Faculty, 21280 Diyarbakır, Turkey; ^2^Department of Anesthesiology and Reanimation, Kilis State Hospital, Kilis, Turkey; ^3^Department of Anesthesiology and Reanimation, Istinye State Hospital, Istanbul, Turkey; ^4^Department of Anesthesiology and Reanimation, Bezmialem Vakif University, Istanbul, Turkey; ^5^Department of Internal Medicine, Trakya University Medical Faculty, Edirne, Turkey

## Abstract

Obesity has a significant effect on the cephalic spread of a spinal block (SB) due to a reduction in cerebrospinal fluid (CSF). SB is controlled by the tissue blood flow in addition to the CSF. Some positions and techniques of surgery used can cause changes in hemodynamics. We investigated effects of hemodynamic changes that may occur during Transurethral prostate resection (TUR-P) and lithotomy position (LP) at the SB level in obese versus nonobese individuals. Sixty patients who had undergone TUR-P operation under spinal anesthesia were divided into a nonobese (BMI < 25 kg/m^2^, Group N) or obese (BMI ≥ 30 kg/m^2^, Group O) group. SB assessments were recorded afterthe LP. SB at 6 and 120 min and the peak SB level were compared between two groups. Hemodynamics were recorded after LP. Peak and 6 min SB levels were similar between the groups, while 120 min SB levels were significantly higher for Group O (*P* < 0.05). Blood pressure (BP) after the LP was significantly higher for Group N (*P* < 0.05). LP and TUR-P increased the BP in Group N when compared to Group O. The increase in hemodynamics enhances the blood flow in the spinal cord and may form similar SB levels in nonobese patients to those in obese patients. However, SB time may be longer in obese patients.

## 1. Introduction

Anesthesia in obese patients has always been considered a significant problem. The decreased volume of cerebrospinal fluid (CSF) in obese patients can cause spinal anesthesia to expand in the cephalic direction [1, 2].

Some positions of surgery and the surgical techniques themselves can cause changes in hemodynamic parameters [3, 4]. The tissue concentration of local anesthetic in spinal anesthesia is controlled by the tissue blood flow in addition to the CSF [5]. Transurethral prostate resection (TUR-P), a surgical treatment used in benign prostatic hyperplasia, is carried out in the lithotomy position [6]. This position meaningfully increases the systolic arterial pressure from the subextremities with the effect of autotransfusion [7]. At the same time, during TUR-P, an entrance of liquid occurs into the system that is proportional to the liquid used, the hydrostatic pressure of the solution, the number of opened venous sinuses, the time of the irrigation, the absorption speed, and amount of irrigation liquid [8]. All these factors cause changes in cardiovascular system.

The purpose of our study is to determine the effects of hemodynamic changes on the level and time of spinal block that may occur during TUR-P in obese versus nonobese individuals.

## 2. Patients and Methods

This study was approved by the Institutional Review Board of Dicle University Medical School and all patients provided informed consent. A total of 60 male patients who had undergone TUR-P operations under spinal anesthesia with an American Society of Anesthesiologists (ASA) score of I or II in the Urology Department were included in this prospective study. Patients with BMIs lower than 25 kg/m^2^ constituted the nonobese group (Group N, *n* = 30) and those with BMIs equal to or greater than 30 kg/m^2^ constituted the obese group (Group O, *n* = 30). The body mass indices (BMIs) of the patients were calculated by dividing patient weight by height squared in meters (kg/m^2^). Neurological disease, deformities of the spinal column, sensitivity to bupivacaine or other contraindications for spinal anesthesia, and skin infection at the site of injection were defined as exclusion criteria

All patients were premedicated with 0.03 mg/kg IV midazolam 30 min before the anesthesia. Patients in both groups were monitored with continuous electrocardiography (ECG), cyclic noninvasive blood pressure (NIBP), and peripheral oxygen saturation (SpO2) in the operating room. All patients were administered 10 mL/kg of lactated Ringer's solution before the spinal anesthesia. Dural puncture was performed at the L3-4 interspace using a 25-gauge Quicke spinal needle in the sitting position. 3 mL of 0.5% hyperbaric bupivacaine was injected over 20 s. Patients were immediately placed in a supine position after the spinal anesthesia and then all patients were immediately placed in the lithotomy position. In this study, the standard lithotomy position was used; both thighs were lifted 90° toward the trunk and the lower legs were hung on poles with ties. The operating table was in the horizontal position. Oxygen was given at 2L/min via nasal cannula during surgery. Analgesia was defined as the inability tosense pinprick. Success of spinal anesthesia was defined as a bilateral T10, sensory block to pinprick within 15 min of intrathecal drug administration. Motor block in the lower limbs was classified using the Bromage Scale [9]: 0 = ability to lift an extended knee at the hip; 1 = ability to flex the knee but not to lift an extended leg; 2 = ability to flex toes only; and 3 = inability to move hip, knees, or toes. Sensory and motor block assessments weremeasured and then recorded at 2, 4, 6, 10, 20, and 30 min afterthe lithotomy position and after intrathecal drug administration at 120 min by an assistant. Sensory and motor block at 6 and 120 min (Marcaine Spinal Heavy, time to onset of effect in minutes: 5–8 min, duration of effect in hours: 2-3 h for urological surgery [10]) and the levels of peak sensory and motor block were compared between the groups.

The patient's systolic blood pressure (SBP), diastolic blood pressure (DBP), mean arterial blood pressure (MAP), heart rate (HR), and peripheral oxygen saturation (SpO2) were measured and then recorded at five-minute intervals from there after the lithotomy position for 30 min and then at ten-minute intervals until the end of surgery. Systolic blood pressure (SBP), heart rate (HR), and peripheral oxygen saturation (SpO2) were compared between the groups. Hypotension was defined as SBP less than 70% of the baseline value or less than 90 mmHg. When hypotension occurred, repeated intravenous ephedrine bolus doses of 5 mg were administered. Bradycardia was defined as HR less than 60 beats/min. If the heart rate was <60 beats/min, atropine (0.5 mg) was administered. Nausea and vomiting events were recorded. Intraoperative nausea and vomiting were treated with intravenous metoclopramide.

The data were analyzed using the SPSS 16.0 for Windows (SPSS Inc., Chicago, IL, USA) packet program. Descriptive statistics of the demographic data and constant variables were indicated as mean ± standard deviation. Data were analyzed using Student's *t*-test depending on the normality of data. Chi-square test was used to analyze incidence data. Parametric repeated data were evaluated by a repeated measures ANOVA test. The value of *P* < 0.05 was accepted as statistically significant.

## 3. Results

No significant differences were noted between the two groups with respect to age, height, duration of surgery, or male/female ratio. The two groups differed with regard to weight and BMI, as expected (Table 1).

**Figure 1:**
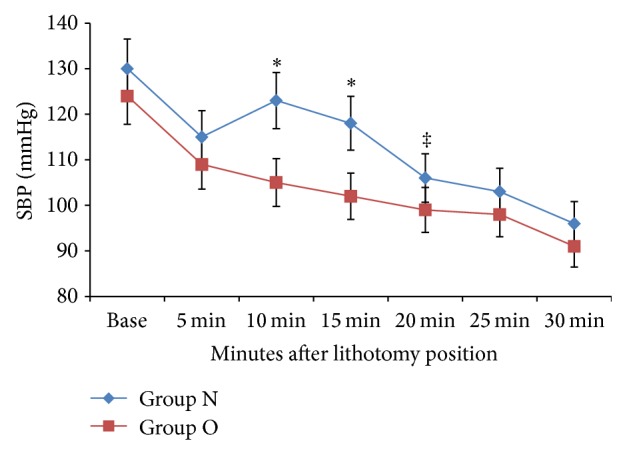
After lithotomy position systolic blood pressure (SBP) (between Group O and Group N); ^∗^ *P* : 0.001 10 min and 15 min,  ^‡^ *P*: 0.05 20 min.

Basal hemodynamic parameters were similar between the groups. Cardiovascular responses are shown in Table 2. In both groups, SBP, 5 min after spinal block, decreased from the baseline value. Systolic blood pressure (SBP) values were measured at 10, 15, and 20 min after the lithotomy position were significant increased for Group N compared to Group O (*P* < 0.001; *P* < 0.001; *P* < 0.05, resp.) (Figure 1). HR values were similar between the groups.

Sensory and motor block levels are shown in Table 3. Peak sensory and motor block levels and 6th minute sensory and motor block levels were similar between the groups. The 120th minute sensory and motor block levels were higher for Group O than for Group N (*P* = 0.017; *P* = 0.008, resp.). No significant intergroup differences were observed with respect to adverse effects (Table 4).

Comparisons made between both groups revealed no significant differences for SpO2 values. No complications were encountered for any of the patients.

## 4. Discussion

Many investigators have stated that obesity affects the time and level of spinal anesthesia [1, 11]. Pressure on the inferior vena cava, which is dependent on increased intra-abdominal pressure, causes distension in the lumbar plexus [12]. This distension can cause a decrease in the volume of CSF, which in turn can affect the level and time of spinal block. In addition, according to different viewpoints, the increased epidural adipose tissue presses on the dural sac in obese patients and causes a decrease in the volume of CSF. Previous studies on obese patients have been conducted with the patients in the supine and lateral decubitus positions [13–16]. However, some surgical positions, such as the head-down tilt and lithotomy positions, may affect arterial tension in the patients. The resulting changes in blood flow can affect the pharmacokinetics of local anesthetics.

In our study, which differed from previous studies, we compared the effects of hemodynamic changes occurring in patients who underwent TUR-P in the lithotomy position on the block levels and times in obese and nonobese patients. In the lithotomy position, a volume of blood of approximately 500–1000 mL passes from the lower extremity to the central circulation as an effect of autotransfusion [7]. Similarly, during transurethral prostate resection (TUR-P), the absorption of irrigation solution in small amounts from the veins in the resection area can reach dimensions that may threaten life depending on the speed of the absorption [8]. In our opinion, blood and irrigation solutions that pass to the central circulation cause fullness in the epidural veins, so that decreases in the volume of CSF and spinal anesthesia time and level may occur in obese patients as well as in nonobese patients.

A study conducted by Miyabe and colleagues [7] showed that SBP increased meaningfully in patients in the lithotomy position compared to the supine position. In our study, the SBP value was meaningfully higher in the nonobese group than in the obese group. Although the difference between the peak block levels was not statistically significant, an unexpected decrease was seen in the obese group. The reason for this may be attributed to the presence of increased intra-abdominal pressure to the inferior vena cava in obese patients and the prevention of venous return. At the same time, the movement of intra-abdominal organs in an upward direction due to the lithotomy position may have increased the pressure on the vena cava and more strongly prevented venous return.

The venous circulation of the prostate is realized by vesicle veins and the internal iliac vein. These vessels drain into the vena cava inferior. In obese patients, abdominal pressure increases due to the weight of abdominal content. Intra-abdominal pressure increases the pressure on inferior vena cava in parallel and decreases the venous return to the heart, thereby resulting in reduced cardiac output [17]. In addition to the lithotomy position, the occlusion force on the inferior vena cava, which is located retroperitoneally, is increased with the effect of gravity due to the weight of the abdominal contents. This increase in the hydrostatic pressure in the inferior vena cava reduces the amount of irrigation solution that joins systemic circulation in obese patients compared to nonobese patients and TUR-P may be less effective in obese patients on hemodynamics.

No statistically significant difference was noted during the 6th minute after the lithotomy position in the sensory and motor block evaluation following spinal anesthesia or in the intergroup comparison of the block peak level. However, previous studies [2, 12] have shown that the volume of CSF decreases due to the increased intra-abdominal pressure in obese patients and it may cause deeper blocks because of the decrease in the dilution of local anesthetic. A study conducted by Carpenter and colleagues [2], conducted using a magnetic resonance monitoring technique, showed that lumbosacral CSF volume is important in the distribution of spinal anesthesia and in the peak action time. The probable reason for our patients' failure to show any difference in block level may be due to the increased arterial pressure leading to fullness in epidural veins in nonobese patients and a decrease in the volume of CSF.

The sensory and motor block levels at 120 min following the spinal anesthesia were statistically higher in the obese patients than in the nonobese patients. In spinal anesthesia, the local anesthetic, which is given at the subarachnoid interval, is absorbed by the veins in this interval and eliminated and diffused from the arachnoid or dural membranes. The elimination of local anesthetics is affected by tissue blood flow, where high blood flow increases the elimination of local anesthetics. Because the flow of blood is much higher at the anterior aspect of the spinal cord, the elimination of local anesthetics is faster [5]. In our study, the low level of sensory and motor block levels at the 120th minute in nonobese patients, when compared to obese patients, may have been caused by an increase in blood flow and a subsequent increase in arterial pressure in the spinal cord and the elimination of local anesthetic.

Previous studies conducted with the use of isobaric bupivacaine in the supine position showed a meaningful expansion in the cephalic direction in obese patients when compared to nonobese patients [1, 11, 13]. However, other studies conducted with hyperbaric bupivacaine in the supine position detected no statistically significant differences in peak sensory and motor block levels and times [14, 15], contrary to our hypothesis. However, studies conducted with methods of monitoring showed that radio opaque materials expand more in the cephalic direction in the supine position and in obese patients when compared to nonobese patients [2, 12], in agreement with the findings of our study.

The volume of CSF may show individual differences and CSF volume is difficult to predict when relying on length, weight, and BMI [12, 18]. In our study, our patients had greater average age and height than those in the other studies [14, 15]. These factors may have caused the difference in the outcomes. In the same study [14, 15], the calculated sensory block time was longer in obese patients. In our study, the sensory and motor block levels were significantly higher in obese patients at the 120th minute. However, another topic which must be taken into consideration is the decrease in the elimination of local anesthetic by decreased blood flow, in addition to the individual differences in CSF volume. In obese patients, increased intra-abdominal pressure also prevents the venous return to the heart in addition to being the reason for the fullness in the lumbar venous plexus due to pressure on the vena cava. It may be the reason for the elongation in block time in obese patients when compared to the nonobese patients.

Consequently, lithotomy position and TUR-P meaningfully increased the systolic blood pressure in nonobese patients when compared to obese patients. The increase in hemodynamic parameters increases the blood flow in the spinal cord and may form sensory and motor block levels in nonobese patients that are similar to those in obese patients. However, the increased blood flow enhances the elimination of local anesthetic and may lead to earlier retreatment time for spinal anesthesia.

## Figures and Tables

**Table 1:** Demographic characteristics of obese and normal weight patients and operation duration.

	Obese patients (*n* = 30)	Normal weight patients (*n* = 30)	*P*
Age, years	62.4 ± 13.0	56.5 ± 16.3	0.290
Body weight, kg	92.9 ± 8.2	68.2 ± 5.6	<0.001^*^
Height, cm	169.3 ± 8.8	174.9 ± 4.6	0.452
Body mass index, kg/m^2^	32.4 ± 3.7	22.3 ± 1.7	<0.001^*^
Operation duration, minutes	41.0 ± 21.4	50.9 ± 23.9	0.151

^**∗**^Extremely significant at the level of *P* < 0.001.

**Table 2:** Hemodynamic data.

Group	Minutes after the lithotomy position						
	Base	5 min	10 min	15 min	20 min	25 min	30 min
Group N							
SBP	130.83 ± 16.9	115.17 ± 20.1	123.61 ± 19.3^*^	118.67 ± 12.7^*^	106.49 ± 16.8^‡^	102.71 ± 15.1	96.31 ± 17.8
HR	90.07 ± 14.3	87.43 ± 12.4	80.23 ± 8.1	75.50 ± 11.8	74.48 ± 10.1	72.36 ± 14.6	70.91. ± 8.4

Group O							
SBP	124.49 ± 18.7	109.49 ± 20.6	104.78 ± 12.3	102.33 ± 15.2	98.87 ± 13.8	97.77 ± 15.8	91.39 ± 14.5
HR	86.45 ± 16.5	83.11 ± 3.7	76.63 ± 12.2	72.27 ± 14.1	70.87 ± 9.3	70.19 ± 10.7	67.66 ± 14.3

Changes in systolic (SBP) and heart rate (HR), in the obese and nonobese groups. ^*^Extremely significant at the level of *P* < 0.001, ^‡^Significant at the level of *P* < 0.05.

**Table 3:** The levels of sensory and motor block.

	Obese patients (*n* = 30), *n* (%)	Normal weight patients (*n* = 30), *n* (%)	*P*
Sensory block			
120th minute level			
T4	0 (0.0)	0 (0.0)	**0.017** ^‡^
T6	7 (23.3)	0 (0.0)	
T8	10 (33.3)	8 (26.7)	
T10	10 (33.3)	14 (46.6)	
T12	3 (10.0)	8 (26.7)	
6th minute level			
T4	0 (0.0)	0 (0.0)	**0.410**
T6	1 (3.3)	0 (0.0)	
T8	3 (10.0)	5 (16.7)	
T10	9 (30.0)	5 (16.7)	
T12	17 (56.7)	20 (66.6)	

Motor block			
120th minute Bromage			
0	0 (0.0)	0 (0.0)	**0.008** ^‡^
1	9 (30.0)	21 (70.0)	
2	15 (50.0)	6 (20.0)	
3	6 (20.0)	3 (10.0)	
6th minute Bromage			
0	9 (30.0)	12 (40.0)	**0.714**
1	16 (53.3)	14 (46.7)	
2	5 (16.7)	4 (13.3)	
3	0 (0.0)	0 (0.0)	

The maximum levels of sensory and motor block			
Maximum Bromage Scale 0-1-2-3, (*n*)	0-3-17-10	0-4-19-7	**0.676**
Peak level of sensory block	T7 (T4–T10)	T7 (T4–T10)	**0.753**

^‡^ *P* < 0.05 (between Group O and Group N) 120th minute the levels of sensory and motor block.

**Table 4:** Incidence of adverse effects.

Adverse effects	Group O *n* (%)	Group N *n* (%)	*P*
Hypotension	5 (16.6)	2 (6.6)	**>0.05**
Bradycardia	2 (6.6)	1 (3.3)	
Vomiting	5 (16.6)	4 (13.3)	
Nausea	7 (23.3)	5 (16.6)	
